# miR-17 regulates melanoma cell motility by inhibiting the translation of ETV1

**DOI:** 10.18632/oncotarget.4147

**Published:** 2015-06-01

**Authors:** Ronit Cohen, Eyal Greenberg, Yael Nemlich, Jacob Schachter, Gal Markel

**Affiliations:** ^1^ Ella Institute of Melanoma Research, Sheba Medical Center, Ramat-Gan, Israel; ^2^ Clinical Microbiology and Immunology, Sackler School of Medicine, Tel Aviv University, Tel Aviv, Israel; ^3^ Talpiot Medical Leadership Program, Sheba Medical Center, Ramat-Gan, Israel

**Keywords:** melanoma, microRNAs (miRNAs), miR-17, ETV1, motility

## Abstract

Melanoma is an aggressive malignancy with a high metastatic potential. microRNA-17 (miR-17) is a member of the oncogenic miR-17/92 cluster. Here we study the effect of miR-17 on melanoma cell motility. Over expression of the mature or pri-microRNA form of miR-17 in WM-266-4 and 624mel melanoma lines enhances cell motility, evident in both wound healing and transwell migration assays. TargetScan algorithm predicts the PEA3-subfamily member ETV1 as a direct target of miR-17. Indeed, a 3–4-fold decrease of ETV1 protein levels are observed following miR-17 transfection into the various melanoma lines, with no significant change in ETV1 mRNA expression. Dual luciferase experiments demonstrate direct binding of miR-17 to the 3′-untranslated region of ETV1, confirmed by abolishing point mutations in the putative binding site. These combined results suggest regulation of ETV1 by miR-17 by a direct translational repression. Further, in both melanoma cell lines ETV1 knockdown by selective siRNA successfully pheno-copies the facilitated cell migration, while overexpression of ETV1 inhibits cell motility and migration. Altered ETV1 expression does not affect melanoma net-proliferation. In conclusion, we show a new role for miR-17 in melanoma, facilitating cell motility, by targeting the translation of ETV1 protein, which may support the development of metastasis.

## INTRODUCTION

Melanoma, a skin cancer that arises from malignant transformation of melanocytes, is the most aggressive form of skin cancer [1]. The melanocytes undergo stepwise transformation to enable the aberrant growth, resistance to apoptosis, highly invasive potential, adhesion, motility and proteolytic capacity, which are all key biological factors in the aggressive clinical course of the metastatic disease (reviewed in [1], [2], [3]). Therefore, delineation of these mechanisms form the basis of understanding the pathogenesis and subsequent development of therapy.

MicroRNAs (miRNAs) are endogenous short noncoding RNA molecules that regulate gene expression. Once processed from the hairpin and loaded into the Argonaute protein of the silencing complex, the miRNAs pair with mRNAs to direct posttranscriptional repression by inhibiting mRNA translation or facilitating mRNA degradation ([4], [5]). The seed region of each miRNA (nucleotide positions 2–8) determines binding to specific sites at 3′-untranslated regions (UTRs) of target mRNAs [6]. miRNAs regulate a variety of biological processes, including cell cycle, differentiation, development and metabolism (reviewed in [7]). Accordingly, they participate in many diseases including cancer ([8], [9]), controlling cancer development, progression and metastasis *in vitro*, *in vivo* and in patients ([10], [11]). The miRNA molecules act either as oncogenes ([12], [13], [14]) or tumor suppressors ([15], [16], [17], [18]) in a tissue dependent matter. This is probably derived from the widely different gene expression profile in each tissue ([19], [20]).

miR-17/92, a polycistronic miRNA cluster that gives rise to miR-17 [21], is mostly considered to play an oncogenic role [22], [23], [12]). Indeed, this cluster is highly expressed in a variety of cancers, including melanoma ([6], [24], [25]). It was reported to regulate normal development and malignant transformation by promoting proliferation, inhibiting differentiation and augmenting angiogenesis ([26], [27], [28]). While it was reported that miR-17 suppresses growth of cervical cancer cells and promotes their apoptosis [29], as well as inhibits the proliferation of some breast cancer cells [30], miR-17 is mostly considered as an oncogene. It increases the invasiveness of breast cancer [31] and prostate cancer [32], as well as the proliferation of pancreatic cancer [33] and prostate cancer [32] cell lines. We have shown that miR-17 enhances melanoma cell proliferation by targeting the ADAR1 protein ([34], [35], [36]). So far, the role of miR-17 in regulation of melanoma cell migration has not been reported.

The ETS (E-twenty six) transcription factor family contains 28 proteins. The ETS domain is the defining characteristic of the encoded proteins, which is composed of ∼85 amino acids and binds to DNA sequences with a 5′-GGA(A/T)-3′ core [37]. The 28 human ETS proteins are clustered into 12 subgroups; one of them is PEA3 subfamily which consists of ETV1, ETV4 and ETV5. This subgroup is known to contribute to various cancer types development (reviewed in [38]).

ETV1 protein regulates many target genes that modulate a variety of biological processes including growth, migration, proliferation and differentiation (reviewed in [38]). Studies suggest an oncogenic role for ETV1 in different cancers, due to its tumor-promoting functional features, for example proliferation of breast cancer [39], proliferation and invasion of prostate cancer [40] [41]. Moreover, in Ewing sarcoma tumors, the EWS gene might be translocated onto the ETV1 gene and the fusion protein exerts oncogenic properties [42].

The current knowledge about the role of ETV1 and mechanism in melanoma is very limited. A recent study showed a small subset of melanomas (5.3%), which over-expresses ETV1 [43]. Another work has identified copy gains of ETV1 gene present in 13% of primary and 18% of metastatic melanomas, which correlated with enhanced proliferation [44]. Whole-genome sequencing in melanoma revealed six distinct re-arrangements involving breakpoints within the ETV1 introns [45], which are associated with ETV1 amplification. Taken together in melanoma, the role of ETV1 and its mode of regulation are still mostly unknown. Here we demonstrate that ETV1 inhibits melanoma cell migration, and further define miR-17 as a direct regulator of ETV1 expression at the protein translation level.

## RESULTS

### miR-17 enhances the motility of melanoma cells

**Figure 1:**
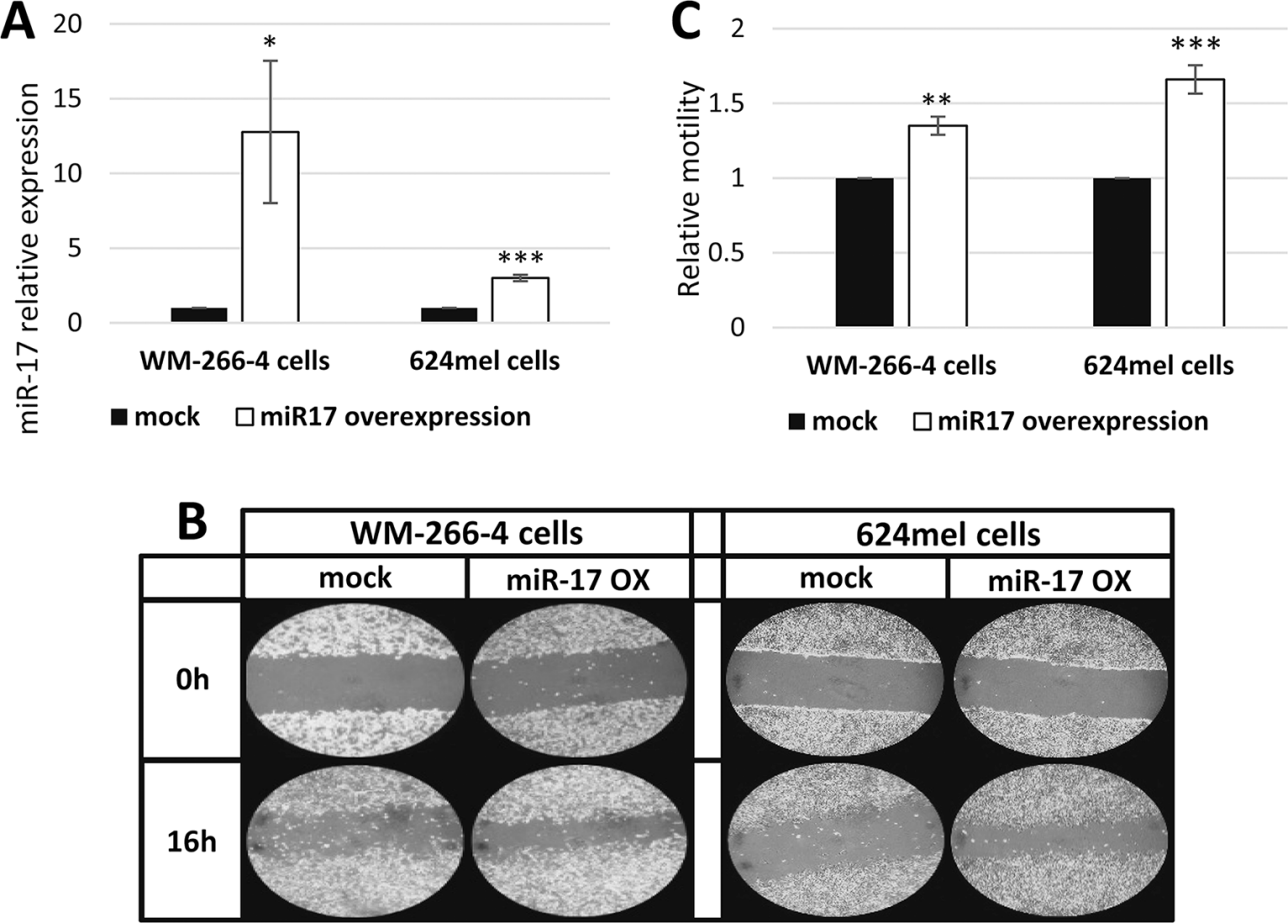
miR-17 enhances the motility of melanoma cells **A.** miR-17 over-expression in melanoma transductants (WM-266-4 and 624mel) was confirmed with qPCR; **B.** Wound healing assay of miR-17 transductants. A scratch was performed after cells reached full confluence and microphotographs were taken at 0 h and 16 h. Figure shows representative microphotographs at x100 magnification; **C.** Wound healing assay analysis. Quantification of the stripped area width was performed with ImageJ. Figure represents the mean of three independent experiments. *denotes *P* < 0.05, **denotes *P* < 0.01, ***denotes *P* < 0.001 (2-tailed *t*-test).

In order to evaluate the functional effect of miR-17 on melanoma cells, pri-miR-17 was cloned and stably over-expressed in WM-266-4 and 624mel melanoma cell lines. An empty vector (mock) served as control. Over-expression of miR-17 was confirmed with qPCR (Figure 1A). The transductant cells were tested *in-vitro* for motility by wound healing assay. Remarkably, miR-17 substantially enhanced the motility of both WM-266-4 and 624mel melanoma cell lines by 35% and 66% respectively, as compared to the control cells (Figure 1B–1C).

### miR-17 enhances the migration of melanoma cells

**Figure 2:**
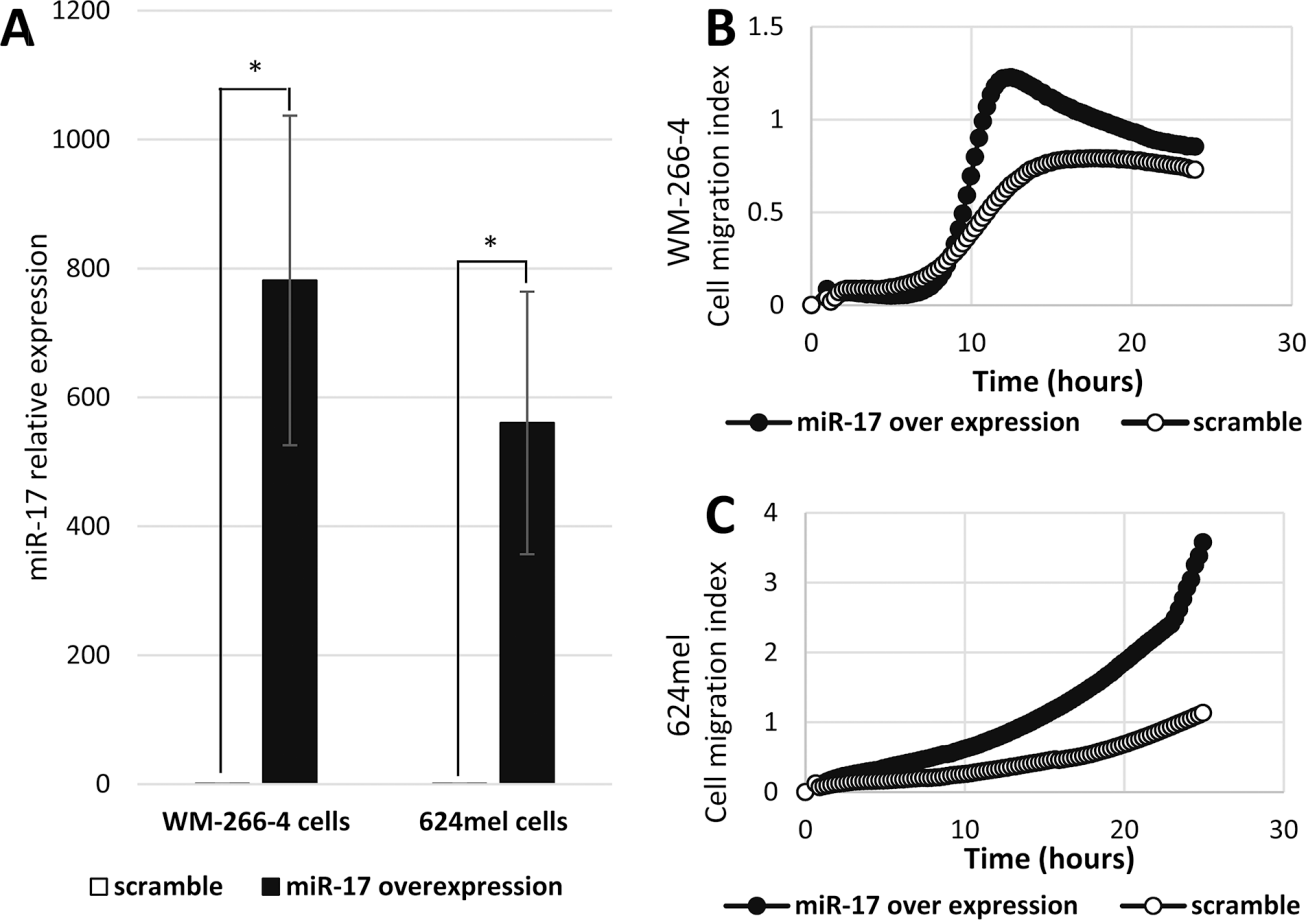
miR-17 enhances the migration of melanoma cells **A.** Mature miR-17 over-expression in melanoma cell lines transient transfectants (WM-266-4 and 624mel) was confirmed with qPCR. *denotes *P* < 0.05 (2-tailed *t*-test). **B-C.** Migration of miR-17 or scramble sequence transfectants was quantified by measurement of electric impedance differences of the transwell, using the xCELLigence RTCA. Figures represent a representative experiment out of three for each melanoma cell line.

In order to test directly the effect of the mature miR-17, as well as to obtain higher expression levels of miR-17, we transiently transfected WM-266-4 and 624mel cells with mature miR-17 or with a scrambled sequence miRNA (scramble), as control. Over-expression of mature miR-17 was confirmed by qPCR (Figure 2A). The transfected cells were tested *in-vitro* for migration through transwell system, using the XCELLigence Real Time Cell Analyzer. Concurring with the wound healing experiments, overexpression of the mature miR-17 substantially enhanced the migratory ability of both WM-266-4 and 624mel cell lines as compared to the control cells (Figure 2B–2C).

### miR-17 is a direct regulator of ETV1

Next we aimed to reveal the mechanism through which miR-17 enhances motility and migration of melanoma cells. Application of the bioinformatics algorithm TargetScan, resulted in a list of potential miR-17 target genes [4]. We selected ETV1 as a possible target of miR-17, due to the fact that its role in melanoma has not been fully elucidated ([43], [44], [45]) and studies of prostate cancer exhibited that ETV1 affects the migratory ability of the cells ([34], [41]). The potential targeting of ETV1 by miR-17 was further validated with two additional independent prediction tools (i.e. miRDB and PITA; data not shown) [46, 47].

**Figure 3:**
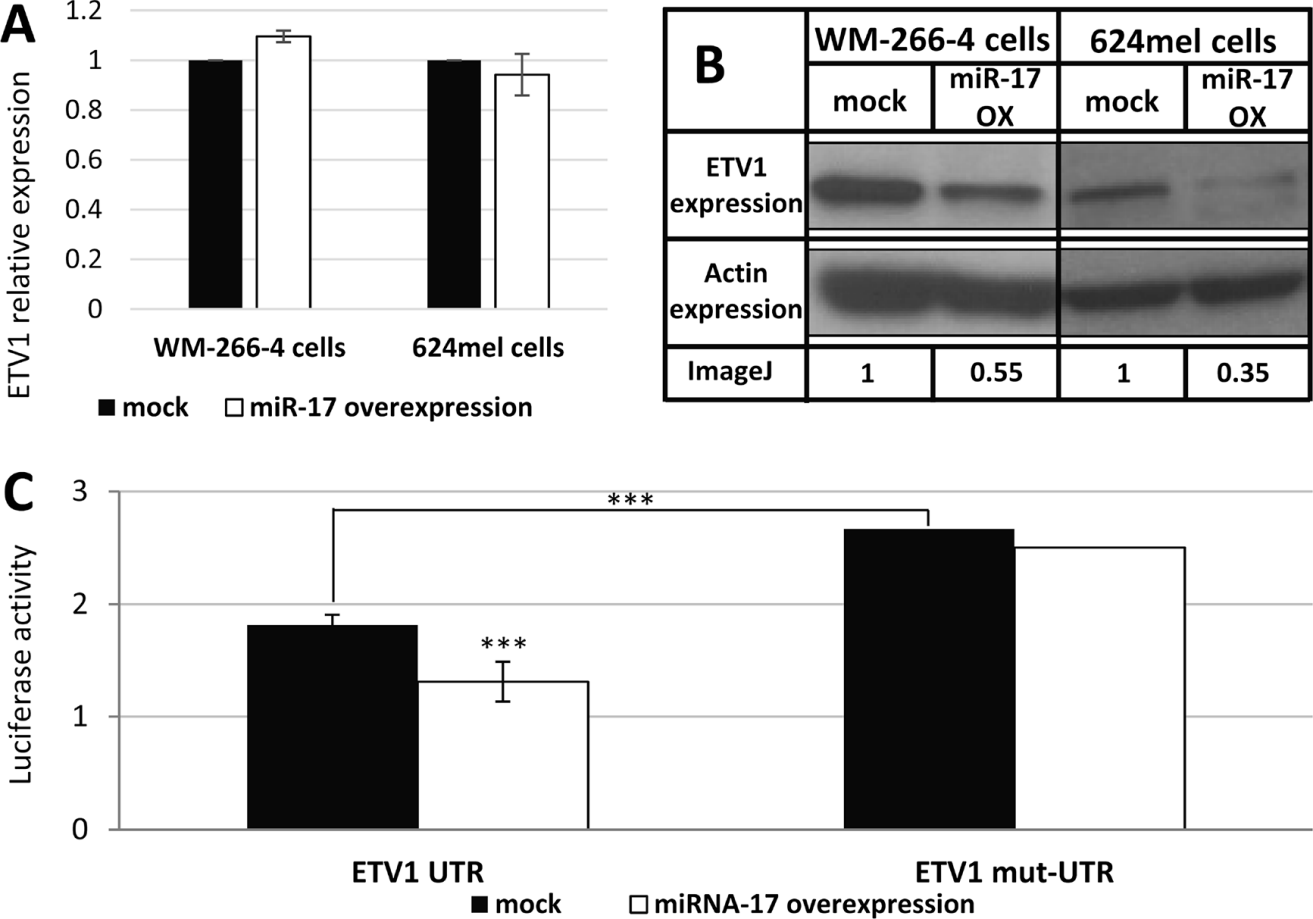
miR-17 is a direct translational repressor of ETV1 **A.** The mRNA levels of ETV1 following transfection with scrambled sequence (mock) or miR-17 in the indicated melanoma cell lines was confirmed with qPCR. Figure shows the mean of 3 independent experiments performed; **B.** ETV1 expression at the protein level was determined by Western blot following transfection with scrambled sequence (mock) or miR-17 in the indicated melanoma cell lines. Actin levels served as control. Normalized densitometry values of ETV1 relative to actin are indicated. A representative blot is shown out of 3 experiments performed; **C.** UTR and mut-UTR denote ETV1 3′UTR segments containing the reference sequence or mutated sequence in the miR-17-binding site, respectively. Those were cotransfected with miR-17 (miR-17 over-expression) or with an empty vector (mock) to 293T cells. Figure shows the mean of 3 independent experiments performed Y-axis denotes normalized Renilla luciferase activity. ***denotes *P* < 0.001 (2-tailed *t*-test).

The effect of miR-17 over expression on ETV1 mRNA and protein levels was tested in both WM-266-4 and 624mel cells. While there were no significant changes in the mRNA levels of ETV1, the protein levels decreased substantially (Figure 3A–3B). Direct binding of miR-17 to the 3′UTR of ETV1 was demonstrated with dual luciferase assay. Indeed, a 48% reduction in luciferase activity was demonstrated following miR-17 over-expression (Figure 3C). Abolishment of the putative binding within the 3′UTR with point mutations significantly enhanced the luciferase activity over basal level (Figure 3C). This is explained by the effect of endogenous miR-17 expression. Importantly, the mutated construct was unaffected by overexpression of miR-17. These combined results strongly suggest that miR-17 directly targets ETV1 by translational repression.

### Knockdown of ETV1 pheno-copies the effect of miR-17 overexpression

**Figure 4:**
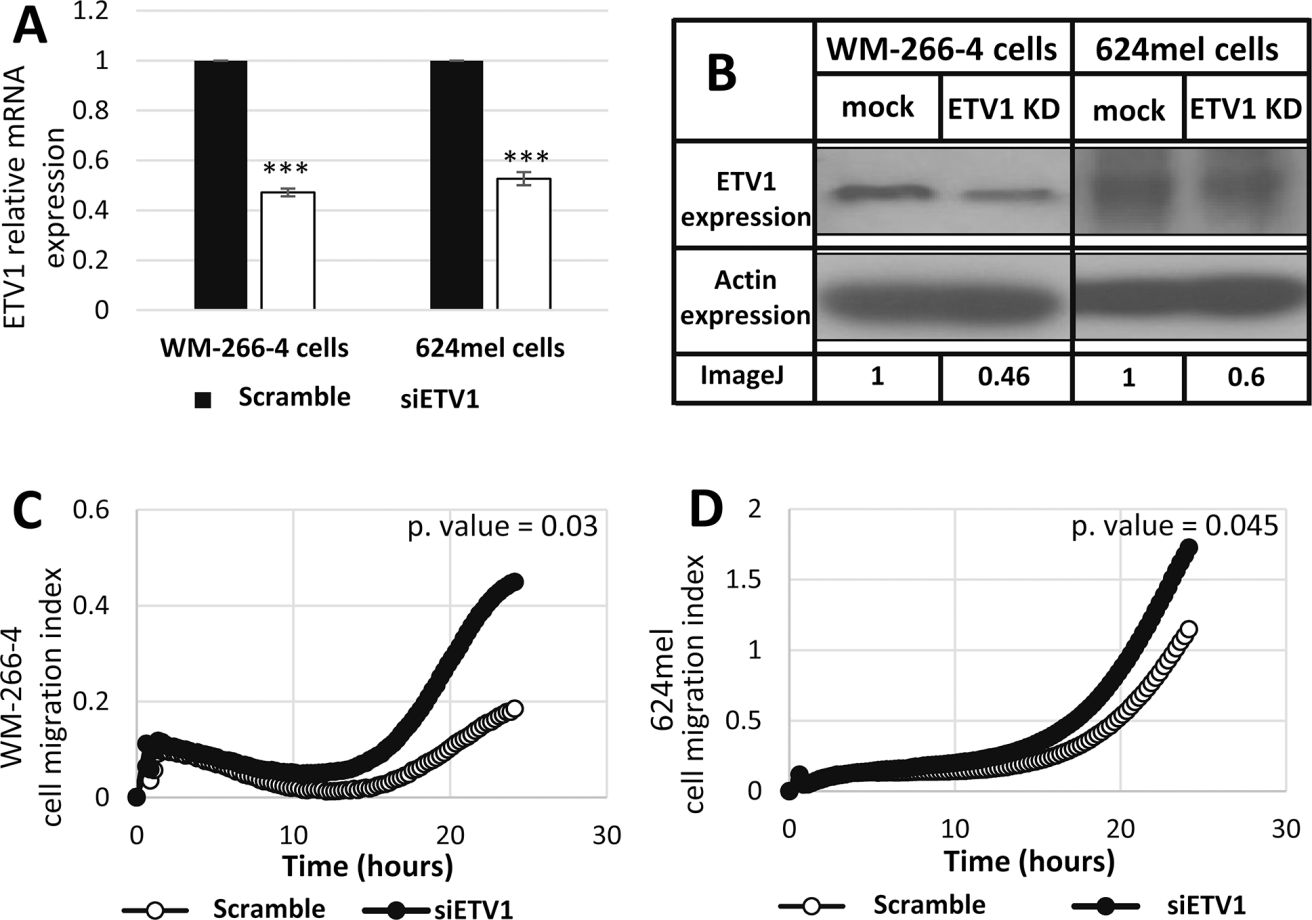
Knockdown of ETV1 expression enhances the migration of melanoma cells **A.** Transfection of siRNA against ETV1 was performed in the indicated melanoma cell lines. mRNA levels of ETV1 was confirmed with qPCR. Figure shows the mean of 3 independent experiments performed; **B.** ETV1 expression at the protein level was determined by Western blot following transfection with scrambled sequence (mock) or siRNA against ETV1 in the indicated melanoma cell lines. Actin levels served as control. Normalized densitometry values of ETV1 relative to actin are indicated. A representative blot is shown out of 3 experiments performed; **C-D.** Migration of transfectants was quantified by measurement of electric impedance differences of the transwell with the xCELLigence RTCA instrument. Figures represent a representative experiment out of three for each melanoma cell line. ***denotes *P* < 0.001 (2-tailed *t*-test).

The expression of ETV1 was selectively knocked down with siRNA in WM-266-4 and 624mel cells. A scrambled sequence siRNA served as control. Knockdown of ETV1 was confirmed with qPCR and Western blot (Figure 4A–4B). The cells were then tested for migration *in vitro* by applying the XCELLigence transwell-based migration assay. Remarkably, knockdown of ETV1 significantly enhanced the migratory ability of both WM-266-4 and 624mel melanoma cell lines, as compared to the control cells (Figure 4C–4D). These results strongly suggest that the effect of miR-17 on migration (Figure 1–2) is mediated by ETV1, which it directly downregulates (Figure 3).

### ETV1 inhibits the motility and migration without affecting net-proliferation of melanoma cells

**Figure 5:**
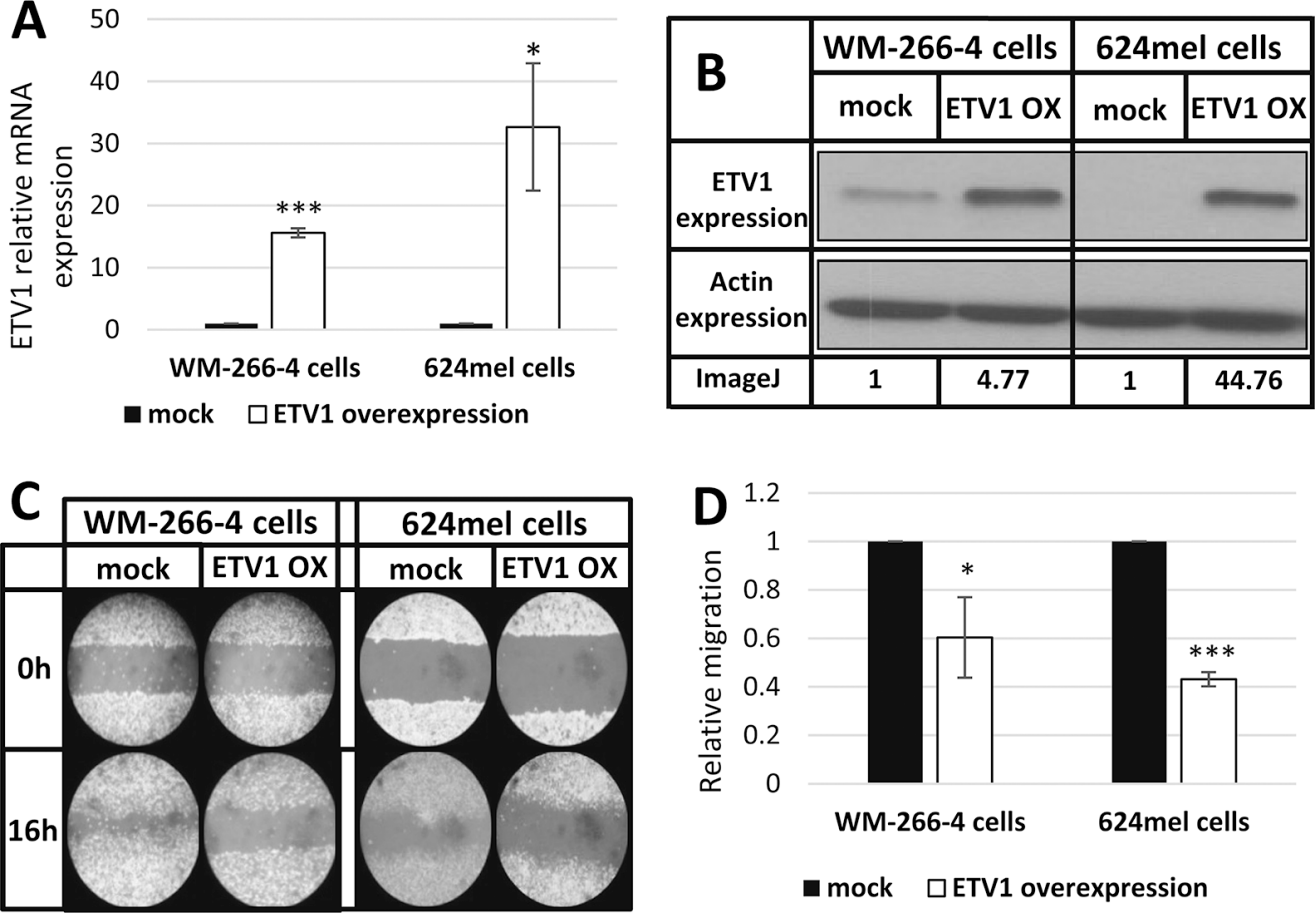
ETV1 inhibits the motility of melanoma cells **A.** ETV1 over-expression in melanoma cell lines transductants (WM-266-4 and 624) was confirmed with qPCR. *denotes *P* < 0.05, ***denotes *P* < 0.001 (2-tailed *t*-test). **B.** ETV1 over-expression of ETV1 transductants determined by Western blot, using anti-ETV1 and anti-actin antibodies. A representative blot is shown with imageJ analysis values of ETV1 relative expression. **C.** Wound healing assay of ETV1 transductants. A scratch was performed after cells reached full confluence and microphotographs taken at 0 h and 16 h. Figure shows representative microphotographs at x100 magnification. **D.** Wound healing assay analysis. Quantification of the stripped area width was accomplished by imageJ. Figure represents the mean of three independent experiments. *denotes *P* < 0.05, ***denotes *P* < 0.001 (2-tailed *t*-test).

**Figure 6:**
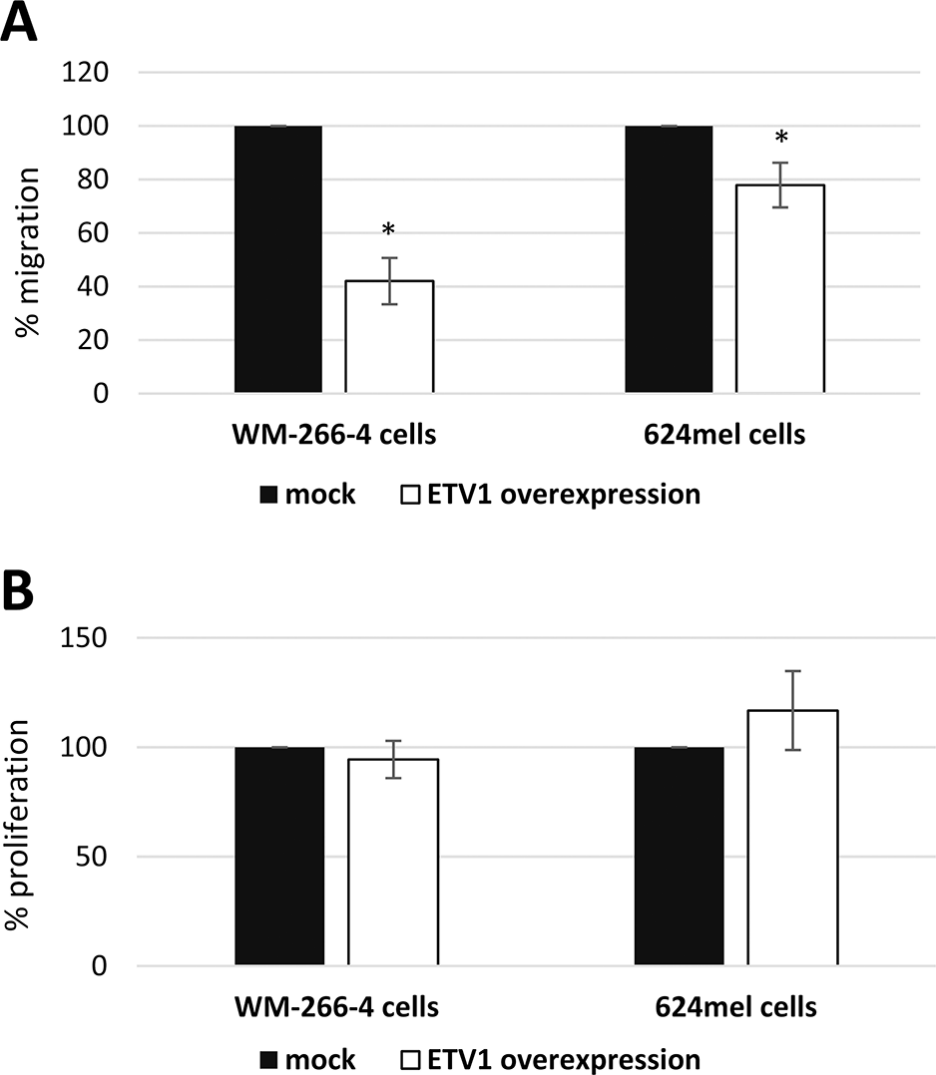
ETV1 inhibits the migration and does not affect net-proliferation of melanoma cells **A.** Migration of ETV1 transductants was quantified with a 12 h transwell assay. Migration was determined with standardized XTT test. The number of mock-transductants was determined as 100%. Figure represents the mean of three independent experiments, each performed in triplicates. *denotes *P* < 0.05 (2-tailed *t*-test). **B.** Net proliferation of ETV1 transductants was quantified with standardized XTT test. The number of cells was determined 48 h after seeding. The number of mock-transductants was determined as 100%. Figure represents the mean of three independent experiments, each performed in triplicate.

To validate the effect of ETV1 on melanoma motility and migration, it was stably overexpressed in WM-266-4 and 624mel cells. An empty vector (mock), served as negative control. Overexpression of ETV1 was confirmed with qPCR followed by Western blot (Figure 5A–5B). Motility was tested with wound healing assays. Remarkably, ETV1 overexpression substantially decreased the motility of both WM-266-4 and 624mel melanoma cell lines by 40% and 57% respectively, as compared to the control cells (Figure 5C–5D). Similar results were observed when the same transfectants were tested for migration in transwell assay. Indeed, ETV1 overexpression significantly inhibited the migratory ability of both WM-266-4 and 624mel melanoma cell lines by 58% and 22% respectively, as compared to the control cells (Figure 6A). In order to rule out the possibility that the observed effects of ETV1 on motility and migration are confounded by enhanced proliferation, the ETV1- and mock-transduced melanoma cells were tested with XTT-based net-proliferation assay. Importantly, ETV1 overexpression has little or no effect on the proliferative activity of melanoma cells (Figure 6B).

## DISCUSSION

miRNAs are recognized for their ability to regulate gene expression and thereby a wide range of physiological and pathological processes, by modulating mRNA stability and translation (reviewed in [4] and [7]). Evidence show that perturbation of miRNA expression patterns contributes to the development of a variety of cancers, including melanoma ([48], [49], [50]). Despite the wealth of information on the oncogenic miR-17 in various malignancies, its role in melanoma is still poorly studied. It was reported that it is overexpressed in some melanoma patients [35], and we have previously demonstrated its proliferative effects in melanoma ([34], [35], [36]). Here we show that miR-17 facilitates melanoma cell motility and migration.

We show that over-expression of miR-17 significantly increases cell motility and migration of melanoma cells (Figures 1–2). Our findings are in line with previous reports of miR-17 affecting migration in other non-melanoma cancers ([31], [51]). Three independent prediction tools identified a single 8-mer conserved binding site of ETV1 as a possible direct target of miR-17. Indeed, miR-17 directly inhibited protein production but did not affect the mRNA levels of ETV1 (Figure 3). Moreover, knockdown of ETV1 with ETV1-specific siRNA pheno-copies the enhanced migration observed by overexpressing miR-17 (Figure 4). As expected, overexpression of ETV1 inhibited motility and migration, but had no effect on net-proliferation (Figures 5–6).

ETV1 is a transcription factor, which has been shown to enhance the migration of prostate cancer cells [40]. The results presented in Figures 4–6 clearly support an opposite, inhibitory, effect of ETV1 on the motility and migration of melanoma cells. Noteworthy, the effect was robust and was demonstrated in two different cell lines, with two different biological assays and using two independent methodologies (knockdown and overexpression). Moreover, as opposed to other malignancies such as breast, prostate and Ewing sarcoma ([39], [42], [40], [41]), in which ETV1 affects proliferation, we demonstrate that ETV1 has no effect on melanoma cell proliferation (Figure 6B). This might be explained through differential gene expression profile between cancers [20]. Such differential gene expression might affect the ETV1-regulated gene repertoire. Our combined results therefore suggest that ETV1 has a suppressive role in melanoma.

A recent study showed a very small subset of melanomas (5.3%) that over-expresses ETV1, but the function was not directly tested [43]. Another report suggested that ETV1 may play a role in melanocyte transformation and melanoma-genesis and has identified copy gains of ETV1 gene present in 13% of primary and 18% of metastatic melanomas. Two cell lines with focal ETV1 amplification demonstrated ETV1 dependency in melanoma proliferation, and ETV1 co-expression with oncogenic mutant NRAS or BRAF demonstrated enhanced proliferation. On the other hand, enhanced, ETV1-dependent, proliferation was also observed in a single melanoma cell line that lacked ETV1 amplification. In our study, ETV1 had no effect on the proliferation of melanoma cell lines, despite oncogenic BRAF mutations V600E (624mel) or V600D (WM-266-4). The differences may represent cell line variability. Importantly, we present evidence that ETV1 suppresses motility and migration of melanoma cells and therefore function as a tumor suppressor. To the best of our knowledge, there is only one report, on fibrosarcoma, that suggests an inhibitory role for ETV1, resulting in increased expression of mRNA of p14ARF [52], a known tumor suppressor which activates p53 [53]. Worth noting, the majority of melanomas in fact lack ETV1 amplification, representing > 80% of the cases [44]. The rate of ETV1 gene loss in melanoma is unknown. Whole-genome sequencing in melanoma revealed six distinct re-arrangements involving breakpoints within the ETV1 introns [45], which are associated with ETV1 amplification. In this report we found evidence for ETV1 downregulation by a post transcriptional mechanism, mediated via miR-17.

A combination of qPCR and Western blot experiments on the miR-17 transductants, indicates that ETV1 is regulated by miR-17 predominantly at the protein translation level. Dual luciferase experiments supported direct regulation. The minor effect on mRNA level of ETV1 following transfection of the mature miR-17 is probably due to the radically higher expression of miR-17. It was demonstrated in gastrointestinal stromal tumors that ETV1 is a target of miR-17 [54]. In this study, we demonstrate new evidence that miR-17 induces melanoma cell motility and migration by targeting directly the ETV1 protein at the protein translation level. We show that ETV1 inhibits melanoma cell motility and migration and functions as a tumor suppressor. These results are expected to contribute to our mechanistic understanding of how metastatic melanoma develops as well as to pointing out on new possible molecular targets for development of future therapeutic technologies.

## MATERIALS AND METHODS

### Cells

The human cutaneous melanoma cell line WM-266-4 was purchased from ATCC, and 624mel was obtained from the NIH (obtained from Dr. Steven Rosenberg, Surgery branch of NCI, Bethesda, MD). All melanoma cultures were grown in RPMI medium supplemented with 10% fetal bovine serum (FBS), 100 μg/ml Pen/Strep, 2 mM L-Glutamine, 25 mM Hepes and 1 mM sodium pyruvate (all from Biological Industries, Kibbutz Beit-Haemek, Israel). Stable transductants were cultured similarly in RPMI, but with the addition of 1 μg/ml Puromycine (Calbiochem, USA). 293T cell line (ATCC, USA) was cultured in DMEM (Biological Industries, Kibbutz Beit-Haemek, Israel) containing 10% FBS, 100 μg/ml Pen/Strep, 2 mM L-Glutamine, 1 mM sodium pyruvate and nonessential amino acids (with final concentration x1).

### Cloning into mammalian expression vector

ETV1 coding sequence, ETV1 3′UTR and pri-miR-17 were PCR-amplified from genomic DNA of melanocytes, applying specific primers (ETV1 coding sequence primers are Fw ATGGATGGATTTTATGACCAG and Rev TTAATACACGTAGCCTTCGTTG; ETV1 3′UTR primers are Fw CAAGTGACAGTCAAGCAGGG and Rev TGTAGGACCCCATCCCAA; miR-17 primers are Fw GCTGAATTTGTATGGTTTATAGTTGTTA and Rev GCACCTTAGAACAAAAAGCACT). ETV1 coding sequence and miR-17 sequences were cloned into pQCXIP vector (Clontech laboratories, Mountain View, CA), by applying enzymatic restriction reaction with the NotI and EcoRI restriction enzymes (New England Biolabs, Ipswich, MA). ETV1 3′UTR sequence was cloned into psiCHECK-2 vector (Promega, Fitchburg, WI), with the XhoI and NotI restriction enzymes (New England Biolabs, Ipswich, MA). Empty vectors (e.g. pQCXIP and psiCHECK-2) were served as negative control. All cloned inserts were fully sequenced (Hylabs Laboratories, Rehovot, Israel).

### Cells transductions

1.5 × 10^5^ 293T cells were seeded in a 6-well plate and cultured overnight in DMEM containing 10% FBS (DMEM/FBS). On day 1, cells were transfected with a mixture of 1 μg GAG-POL, 1 μg Envelope, 2 μg of each of the pQCXIP constructs (ETV1, miR-17 or empty vector) and 6 μl of Turbofect reagent (Thermo scientific, Waltham, MA). After six hours of incubation at 37°C, the cells were washed and re-cultured in fresh DMEM/FBS. On day 2, 1.5 × 10^5^ melanoma cells were placed in each well of 6-well plates and cultured overnight in RPMI containing 10% FBS (e.g. T2). On day 3, the melanoma cells were infected with 6ml of 0.45 μm-filtered virion-containing medium of the 293T cells. After incubation at 37°C for 6 hours, the infected melanoma cells were washed and re-cultured with fresh T2. The aforementioned infection procedure was repeated the next day on the same melanoma culture. 48 hours after the second infection, selection was performed by addition of 1 μg/ml puromycine into the culture medium.

### Cells transfections: miR17 or siRNA of ETV1

1 × 10^6^ melanoma cells were seeded in 100 mm plate and cultured in T2. On day 1, cells were transfected with a mixture of 20 μl Jet-prime reagent (Polyplus, Illkirch, France), 500 μl of Jet-prime buffer and either 10 nM of miR-17 (Sigma-Aldrich, St. Louis, MO), 5–10 nM of ETV1 siRNA (Sigma-Aldrich, St. Louis, MO) or same concentration of scrambled sequence siRNAs respectively. After incubation at 37°C overnight, the transfected melanoma cells were washed and re-cultured with fresh T2. Functional assays were applied on the cells 48 hours post transfection.

### RNA isolation

Total RNA was isolated with Tri Reagent (Sigma-Aldrich, St. Louis, MO) according to the manufacturer's instructions.

### Real-time PCR analysis

First, cDNA of genes was generated by transcriptor universal cDNA master kit (Roche, Penzberg Germany) and cDNA of miRNAs by miRCURY LNA™ universal cDNA synthesis kit (Exiqon, Vedbaek Denmark) according to the manufacturer's instructions.

Gene expression was measured with the SYBR green I master (Roche, Penzberg Germany) and gene-specific primers. For miRNAs detection, the SYBR Green master mix and specific-miRNA LNA primers were used according to the manufacturer's instructions (Exiqon, Vedbaek Denmark). The real-time PCR (qPCR) reactions were normalized to GAPDH or SNORD48 endogenous control respectively.

Detection was carried out using the LC480 qPCR machine (Roche, Penzberg Germany) according to the manufacturer's guidelines, followed by melting curve analysis at the end of the run.

### Western blot

Lysates of 5 × 10^6^ cells were analyzed by SDS-PAGE. Western blot for ETV1 (Abcam, Cambridge UK) and β-actin (MP Biochemicals, Santa Ana, CA) with specific antibodies, which was developed with standard ECL reaction, as previously described [34].

### Wound healing assay

5 × 10^5^ melanoma cells were seeded in triplicates in a 6-well plates and cultured for two days to reach full confluence. Cell monolayers were scratched (“wounded”) by dragging a 1 ml pipette tip across the well followed by wash with PBS in order to remove cell debris. Images of the scratch were taken at 0 and 16 h with the Olympus CKX31 microscope (magnification x10) and camera (Olympus, Tokyo Japan).

### xCELLigence real time cell analysis (RTCA-DP) migration assay

8 × 10^4^ melanoma cells were seeded in duplicates into the upper wells on top of the 8 μm pores membrane of a CIM-Plate 16 (ACEA Biosciences, San Diego, CA), in RPMI. The lower well contained the same medium supplemented with 10% FBS and 25 mM Hepes. During 24 h of incubation at 37°C, the xCELLigence RTCA quantified the migration by measurement of electric impedance differences of the membrane every 15 minutes (cell index). Cell index ≥ 0.2 is considered to be active cell migration.

### Transwell migration assay

Migration was tested in modified Boyden chambers. Briefly, 2 × 10^5^ melanoma cells were re-suspended in RPMI containing 0.1% FBS and seeded in triplicates into the upper wells of a 8 μm pores Transwell migration system (Greiner bio-one, Vilvoorde Belgium). The lower well contained the same medium supplemented with 10% FBS and 25 mM Hepes. After 12 h of incubation at 37°C, cells in the upper well, which did not migrate, were removed using cotton swabs. The number of cells that migrated through the membranes was measured by standardized XTT staining (Biological-Industries, Kibbutz Beit-Haemek Israel), according to the specific regression equation that was determined for each cell line tested.

### Net cell proliferation

5 × 10^3^ melanoma cells were seeded in triplicate wells in a 96F-well microplates. Net-proliferation was determined by XTT colorimetric assay (Biological-Industries, Kibbutz Beit-Haemek Israel), according to manufacturer's instruction. Following background subtraction, the O.D. values were transformed into viable cells counts according to the specific regression equation that was determined for each cell line tested.

### Luciferase reporter assay

10 × 10^3^ 293T cells were seeded in sixplicates in a 96 well plate. The cells were co-transfected with 1 μg of psiCheck2-ETV1 3′UTR (e.g. ETV1 UTR) or psiCheck2-ETV1 mutated 3′UTR (e.g. ETV1 mut-UTR) and 0.1 μg of the pQCXIP-miR-17 (e.g. miR-17) or pQCXIP-empty vector (e.g. mock) as control. Cells were harvested 48 hours after transfection and assayed with Dual Luciferase Reporter Assay System (Promega, Fitchburg, WI) according to the manufacturer's instructions.

### ImageJ software

Wound healing images were analyzed by applying the Rectangular tool of ImageJ. Western blot images were analyzed by applying the Straight tool of ImageJ [55].

### Statistics

Data were analyzed by applying the unpaired 2-tailed and paired 1-tailed Student's *T* tests. *P*.value ≤ 0.05 was considered significant.
